# Academy of Stomatology

**Published:** 1900-11

**Authors:** 

**Affiliations:** Academy of Stomatology


### ACADEMY OF STOMATOLOGY.

A regular monthly meeting of the Academy of Stomatology was held at the rooms of the Academy, 1731 Chestnut Street, Philadelphia, on the evening of Tuesday, April 24, 1900, Dr. J. T. Lippincott, President *pro tem.*

A paper entitled "Extraction of Living Pulps from Teeth under the Influence of Cocaine Anæsthesia produced with the Aid of Mechanical Pressure," was read by Dr. Wilson Zerfing.

(For Dr. Zerfing's paper, see page 581.)

### DISCUSSION.

*Dr. Huey.*—I do not have many opportunities to try this experiment, as I do not have many pulps to destroy. Probably three or four times during the past year I have tried the cocaine method, and very successfully. I have not been able to make the operation absolutely painless, as Dr. Zerfing has, but have always inflicted more or less pain, and only resorted to the method in order to accomplish the work at a given time. Dr. Zerfing's reports of success will induce me to experiment further in this line.

I should like to ask Dr. Zerfing if there were any apparent reasons for the failures in the three cases that he cited; also whether his observations were made in private practice.

*Dr. Zerfing.*—In one of them there was none. The patient would submit to almost anything, but the pulp simply would not submit; in other cases the patients would object to almost anything; in still other cases there was no apparent cause that could be discovered. In some cases, where failure might have been expected, we found it very successful, even with sensitive patients. I do not think the opportunity would come in private practice to get that number of cases in a reasonable length of time.

*Dr. Gaskill.*—I notice that a number of these cases that Dr. Zerfing has cited were in comparatively young persons. I wonder if he found it more successful in these patients of sixteen, seventeen, or twenty years of age, and what has been his experience with older persons.

Recently I was very anxious to remove the pulp from a lower first molar, in a man about thirty-eight years old. I had a rubber dam on, put cocaine in, tried to force it in, and worked an hour and a half. Then I put in a little arsenic and brought it out at once.

*Dr. Jack.*—I would state that I have not pursued this method. I have, indeed, not tried it. My usual method is to instil cocaine, or to use cataphoresis. Where I use instillation a rubber dam is placed and a saturated solution of cocaine hydrochlorate applied. I fill the cavity with the saturated solution. In the course of a few minutes I am able to insert the broach and commence the instillation of the cocaine to the deeper parts of the tissues. I proceed by that method to the root of the tooth, until I am able, in a short time, to reach the apex of the tooth and remove the pulp. I might cite one case that I had two or three years ago. A patient came to me from a distance with a pulp fully exposed. I recognized that by treating her in the ordinary way with arsenic I would keep her in town for several days. In less than half an hour, by the method mentioned, I not only removed the pulps from both roots, but filled the roots with oxychloride of zinc, and the same day I filled the external cavity and sent the patient away. Repeatedly since I have done the same thing. In a number of instances, however, I have felt it necessary first to devitalize a portion of the pulp with arsenic, making but one application, and desensitizing the remainder of the pulp by instilling cocaine as related. Generally, however, I prefer cataphoresis, occupying perhaps fifteen minutes in the process of desensitizing the entire pulp.

I had a case a short time ago, where it was necessary to crown two centrals, two laterals, and one bicuspid. In this case the pulps were not exposed, but the teeth had been abraded very nearly to the gum. In one case the resistance to the current was so great that only one-fortieth of a milliampère of current was passing.

The surface of the dentine was so extremely dense, on account of the abrasion, that after trying to introduce the cocaine I had to drill some little distance below the surface and recommence the application of cocaine by cataphoresis. In the course of eighteen minutes the pulp was desensitized to the apex of the root and removed. It was similar in each of the other cases, the time consumed being about fifteen minutes. In no case was there any irritation whatever from the current. In one or more cases it was found that the pulp was not anæsthetized to the apex of the root. The remaining portion was then anæsthetized by instillation, by filling the pulp-chamber with cocaine and conveying it by the ordinary Swiss broach to the apex of the root.

I believe that in the three cases which Dr. Zerfing found to resist the application of the cocaine by pressure I should at once have applied the cocaine by means of cataphoresis, and I think that I should certainly in a few minutes have been entirely successful in producing anæsthesia.

*Dr. James Truman.*—I have nothing very practical to say on this subject. I have had something to do with the observation of some of the cases in the clinic of the University of Pennsylvania, and have tried the experiment there myself several times with varied success. Those cases in which it seemed to me it failed were those in which arsenic would probably fail, and were cases in which the pulps were excessively irritated. In his report Dr. Zerfing mentioned such cases as, for example, pulp-nodules, where there is almost always a degree of inflammation. I do not know whether in his experiments he has tried chloroform instead of alcohol. I would suggest the experiment to him. Chloroform, it seems to me, would give a better result than alcohol, though I have not tried it. Sometimes with alcohol I could work in through the pulp-chamber and get beyond it, but beyond that was sensation which required repeated effort.

I have myself no doubt about the operation; it is a very simple one, that can be and ought properly to be applied in place of arsenic in many cases. I do not think it can always be used, but probably as often as arsenic can be.

*Dr. Head.*—I have been very much interested in the extraction of the pulps without the aid of arsenic for some years, but unfortunately in my hands arsenic has not seemed to give the satisfactory results that many of my brother practitioners have been able to get, although I have frequently been told by others that they were able to kill the pulp by arsenic without any pain whatsoever. The most troublesome pulps were those in which congestion had been caused by pulp-nodules, and when I first began to practise I found arsenic unable to cope successfully with this condition. I later on tried cataphoresis, but the method which I had used, and which I now use very frequently, seems, in the greater number of instances, to have given the best results. I drill into the tooth as far as I can without giving the patient too much pain, and then fit the nozzle of my hypodermic syringe into this opening by means of a packing of rubber dam, so that when I inject cocaine solution into this opening the full pressure can be obtained. While the patient is under the influence of nitrous oxide gas I drill through into the pulp-chamber and inject about one to two minims of a ten to fifteen per cent. cocaine solution. When the patient recovers consciousness the pulp is usually entirely insensible. I make another injection, in order to be doubly sure of absolute anæsthesia; then I open the tooth and remove the pulp in the ordinary way. I have done this with molars, because the rapid extirpation and removal of the filaments of pulps in the roots seemed impossible under gas, but with the centrals and laterals and cuspids, and even the first and second bicuspids, I have found the rapid extirpation of the pulp under gas to have given excellent results. It has only been within the last month or two that I have used the method spoken of this evening by Dr. Zerfing, and while I have not been able to secure absolute freedom from pain, still I must say it is an extremely useful method, and one which, when it has been perfected, will give as good results as any one method that we have ever had. With all methods there will certainly be a number of failures. The rhinologists and other specialists of the medical profession who use cocaine find certain patients for whom cocaine is inactive, and I have patients for whom I have injected cocaine into the pulp with pressure, and it has remained absolutely unaffected by it, although the patient had some slight systemic effects from the cocaine, showing conclusively that the cocaine had entered the tissue.

*Dr. Kay.*—I have been trying this method for some time, and when I used alcohol I did not always have very good results, but sometimes I did have. It was suggested to me to use chloroform, and since then I have been using chloroform and cocaine, and in every case I have been able to take the pulp out absolutely without pain and in most cases within two minutes from the starting of the operation, which I think is quite a success.

*Dr. Gaylord.*—About two years ago I experimented with this method, as sold in the West as a secret. It was only a short time after that an opportunity presented itself for trying it, and while I did get the pulp out, it was not a painless operation by any means, and it required, I think, three applications.

The next case was perhaps more successful, but not entirely so, and then I thought I would try another scheme. With a fine sand-paper disk I sharpened the point of a hypodermic needle as finely as I could. I took off part of the bevel, so that the point of the needle would not have to be inserted deeply under the pulp before the orifice would be entirely embedded, and so that the solution could go directly into the pulp. Once in a while I find a case where the hypodermic cannot be inserted without any previous application. In such case I make the application of cocaine and alcohol, and that will, as in every case I thus far have had, obtund the pulp sufficiently to insert the point of the needle. I find it a very easy matter to inject the cocaine into the pulp. I think it will be the experience of all those who try it that cocaine will penetrate the pulp with very much less force than it will penetrate the gum tissue, and the solution I use is the one for which I believe we are indebted to Dr. Huey,—cocaine hydrochlorate, ten grains; carbolic acid, ten grains; atropia, one-fifth grain; ten minims of one per cent. nitroglycerin solution; water, two fluidounces. This solution I find is almost perfection in the way of a local anæsthetic. I inject it into the gums and wherever I have had occasion to use cocaine, and never yet have I had any ill-action from the cocaine, either sloughing of the gums, paroxysms, or heart trouble. In cases where teeth are cut off for crowns, if one hesitates to drive in the orange-wood stick, the use of the needle can very easily be brought into play. Insert the point of the needle immediately after the tooth has been excised and inject, and there will be no trouble in extirpation. In every case where I have used the needle the pulp has been removed painlessly, and I consider it very much better than the use of arsenic, even if the first puncture of the needle should cause pain. In using arsenic you are always liable to have a severe toothache in a couple of hours. So far as my experience goes, if you once get the point of the needle in, you can always get the pulp out without much pain.

*Dr. Huey.*—That formula ought to have quotations around it. It is a published formula which I repeated.

*Dr. Jeffries.*—I fortunately do not treat many pulps, and I have not had a very extended experience with the use of cocaine, except that had some months or a year ago, with the aid of cataphoresis, which I have largely abandoned, because of want of success with it, which is perhaps due to my inability to use it properly. I have extracted, however, without pain in a few cases with the aid of the cataphoretic current and cocaine, but my experience is not very extended in that line. I still rely more upon arsenic than upon cocaine.

*Dr. Jameson.*—I have had very little experience in the use of cocaine, except with the aid of cataphoresis, which generally proves satisfactory, if we take the time for it. I almost always use arsenic, and I very seldom have a patient complain of any pain. I make my application, see my patient the next day, remove the application, and make a second. I have learned by experience not to apply arsenic, however, to a pulp that is inflamed. If a patient comes to me with a pulp exposed and inflamed, I first soothe the pulp, even if it take two or three days, and after that I apply my arsenic. If properly sealed, my experience is that there is little or no pain. When I have occasion to destroy a pulp that has not been exposed, I expose it with a drill and make my application, and it is very seldom that the patients say that they have had any pain with the application. That does not agree with the experience of Dr. Zerfing and a great many others, but that is my experience. I always see the patient the following day, and if necessary make a second application.

*Dr. Register.*—The paper we have listened to this evening is one that will probably cause a great many to investigate the subject much more thoroughly than heretofore. We should remember that we get a complete circulation of the blood through the pulp. In the application of arsenic, the reason we get pain is because we produce pressure, which may produce stasis of the circulation in the blood-vessels while the nerve circuit is still open. The only way to reduce pain in a condition of this kind is to resort to force of some character, or to wait until the neurotic energy is lost. I have had some little experience with the use of cocaine, both by cataphoresis and by forcing it into dentine and into pulps of teeth. I have used it in four solutions, all of which I can depend on,—

one with alcohol, one with chloroform, one with alcohol and chloroform mixed, and one with fifty-per cent. solution of sulphuric acid. Cocaine mixes with all of these solutions very beautifully. With fifty per cent. of sulphuric acid it makes a very oily-looking mixture and is one of the best means I know of for the reduction of hypersensitive dentine. The sulphuric acid and its effects should be removed before filling. Of the solutions for pulp removal, I prefer the alcohol. I have in all cases succeeded in removing the pulp at the same sitting, but always have produced some pain. In the application of arsenic, the circulation in the tooth is suspended; and you can get no further influence of any medicinal agent by simple contact other than through a soaking-in influence, which is very slow. In these cases I have used with success a seventy-five per cent. solution of nitrate of silver. I hold it against the exposed portions for perhaps a fraction of a minute, and as it is a very strong coagulant of albumin, it will enter the pulp for quite a distance. You can now insert the point of a hypodermic syringe loaded with cocaine solution and complete the operation under local anæsthesia.

*Dr. T. V. Smith.*—I have on record twenty cases in which I have devitalized the pulp by means of cocaine and pressure, and I have found that the age has something to do with it; at least, such is my experience. In patients from twelve to fifteen years of age, the pressure seems to cause more pain, and in one case out of the twenty I could not obtain anæsthesia on account of the great pain from pressure. In that case I used arsenic, and on opening into that pulp I found the pulp-canal was quite large at the foramen; it bled considerably. In the other younger patients four were between fourteen and seventeen; in all of these there was more sensation than in the other cases, but beyond that age I found there was very little or no sensation whatever upon pressure or upon removal of the pulp.

There must be a good exposure, and all blood must be cleared away. There must be no hemorrhage while the operation is in progress. In one case I had of a molar, there was a slight exposure at the anterior cornu, and by three minutes and a half of pressure with cocaine in acetic ether, applied upon a little piece of spunk, I succeeded in anæsthetizing that pulp completely and in removing it.

I have taken a little cocaine hydrochlorate and mixed it with a little water in order to make a thick paste. I placed a small particle of this paste over the exposure, saturated a piece of spunk with acetic ether, and applied this over the cocaine paste. Then, with soft vulcanizable rubber placed over the entire floor of the cavity, I made pressure with an instrument a little smaller than the opening of the cavity. For the anterior single-rooted tooth a pressure of about two minutes was sufficient; for a molar tooth, three and a half or four minutes. The hemorrhage seemed to be very slight, with the exception of one case, where the blood spurted from the canal quite freely. That was stopped with a little twenty-five per cent. pyrozone on cotton. The after-results in all cases have been very satisfactory. Where I have been compelled to, I have filled the root and crown at the same sitting, with good results.

*Dr. William Trueman.*—The question before us to-night is whether it is better to extirpate a live pulp wholly or partially anæsthetized, or one that has been devitalized and has severed its connection with the surrounding tissues. Is the danger of the devitalizing agent passing beyond the apex of the root greater than that caused by leaving some portions of the tissue in the pulp-cavity, owing to the impossibility of thoroughly removing from it a pulp that still retains its normal connection with normal tissue? It is generally conceded that the pulp is not a mere occupant of the pulp-chamber, but that it is intimately connected with the tubules and apical tissues, and that upon devitalization these connections are severed, and it is possible thoroughly to remove the pulp. Now, can we do it when the pulp is in a normal condition? The possibility of doing it practically painlessly has been from time to time demonstrated. I have done it frequently, and I have known others who have. Many years ago a professional acquaintance said to me that he never used arsenic. He turned a little bur at the end of a Williamson broach, and when he reached the apex with it he cut off the pulp and removed it without, as he said, any more pain than ordinarily given by arsenic. As time went on quite a number of his patients came to me, and I was interested in getting their side of the story, which thoroughly coincided with what he told me. But the real question, everything considered, is whether it has any decided advantages over the devitalizing of the organ before it is removed. Many years ago I attempted to treat a pulp, and was not successful in obtunding sensibility. The patient called on Dr. Thomas to have the tooth removed. He recognized that it was a valuable tooth, and suggested pulp-extirpation under gas, which was accomplished. He sent the patient back to me. So far as could be seen, the pulp was thoroughly extirpated and the canal filled with a little cotton and creosote; but still the patient continued to suffer pain, and I was unable to relieve it. He passed into the hands of a friend of mine, and finally that tooth was extracted. We supposed that a little portion of the broach had passed into the apex, and we expected to find it there, but such was not the case. The tooth was tolerated for a year, and was a constant source of annoyance, with no apparent reason for the extreme sensitiveness. The same thing has happened under cataphoresis, as related at the last meeting of the New Jersey State Dental Society. It is a many-sided question, and one which requires a good bit of experimentation before we can decide upon any one method for all cases, though meanwhile each may have its place. I think the essayist should be commended for his experiments and thanked for having brought this subject before us this evening.

*Dr. Schamberg.*—The discussion has brought a thought into my mind as to whether anæsthesia is entirely due to the cocaine, or whether it is due to pressure. In the first place, the application of cocaine may allow for a certain amount of pressure, but if you press upon a vascular part, enclosed in a bony cavity, you will impede the circulation, and I doubt if any cocaine is absorbed after pressure has once been applied. I do not think that sufficient cocaine can be taken up by the pulp to thoroughly anæsthetize it by simple contact. After pressure is once applied I believe the anæsthesia to be due to temporary paralysis of the nerves, which permits the extraction of the pulp. If that be true, it is probably a better method than the injection of cocaine directly into the pulp, because, as suggested, a certain amount of cocaine can be carried beyond the apex of the root by injection. The same is true of the application of arsenic. Arsenic is absorbed very readily by the tissues, and if carried beyond the apex of the root, the destruction of apical tissue will naturally take place. I think, therefore, that any method which allows for the extraction of the pulp, and in as normal a condition as possible, without the use of any substances which will be taken up by the apical tissue, is the method that should be adopted. The method that Dr. Head has carried out under nitrous oxide is a very admirable one.

*Dr. Nickell.*—It has been my duty to have charge of an institution close to the city, where I have a large number of people to look after. I have used this pressure method for the last year, while going over the district, and I am very much pleased with it. I go there once a week, and could not possibly use arsenic unless I could see the patient within a short time. Sometimes the pulp will come out without the blood being compressed out of it, and after removal, in some cases, there is excessive hemorrhage. This is stopped by pyrozone or sulphuric acid, and the canal filled with oxychloride or gutta-percha.

*The President.*—Dr. Zerfing, will you close the discussion?

*Dr. Zerfing.*—I do not know that I have anything in particular to add. All of the failures were in young patients. I do not know that advanced age would be any factor against the success of the treatment. I have not found it so. I have not tried chloroform in place of alcohol, but from the experiences here given it seems to be successful.

As to the precise time required for the anterior or posterior teeth, I would say that I have found the time would vary from half a minute to five, six, eight, or ten minutes in either anterior or posterior teeth. Some anterior teeth require from five to ten minutes sometimes, but it is not the average. I think the average time is two to three minutes.

If in devitalizing pulps they would be affected by the arsenic just to the apex, all would be well, but if it goes beyond, we all know what may result.

I wish to express my thanks for the kind manner in which the paper was received.

Otto E. Inglis,
*Editor Academy of Stomatology.*

